# Exploring midwifery students’ experiences of clinical training in planned home birth: A qualitative study

**DOI:** 10.18332/ejm/211971

**Published:** 2025-11-27

**Authors:** Trinidad Maria Galera-Barbero, Vanesa Gutierrez-Puertas, Alba Sola-Martínez, Lorena Gutiérrez-Puertas

**Affiliations:** 1 Department of Midwifery, University Hospital Torrecárdenas, Almería, Spain; 2 Department of Nursing, Physiotherapy and Medicine, University of Almería, Almería, Spain; 3 Department of Midwifery, Hospital La Inmaculada, Almería, Spain; 4 Research Group PAIDI-HUM 061 “Experimental and Applied Neuropsychology”, University of Almería, Almería, Spain

**Keywords:** birth experience, midwifery student, education, simulation, planned home birth

## Abstract

**INTRODUCTION:**

In Spain, midwifery students receive limited education on and remain unfamiliar with the physiology and safety of planned home births. The aim of the study was to explore midwifery students’ experiences of clinical training in planned home birth and how to improve the quality of care based on identified barriers.

**METHODS:**

A descriptive qualitative study was carried out. This research was conducted at the Nursing Faculty of the University of University of Almeria, involving 13 midwifery students. Data were collected using semi-structured interview between December and April 2025. Data were analyzed using thematic content analysis and themes generated that addressed the research objective.

**RESULTS:**

A total of 13 midwifery students enrolled in the education program. Two main themes were developed that reflect the meaning patterns constructed through the data analysis: 1) ‘Attend a planned home birth’, with the subthemes ‘Fear of giving birth outside delivery’, ‘The unknown physiological process’ and ‘Recognizing stereotypes around the naturalization of birth’; and 2) ‘Promoting resources that integrate planned home birth’, with the subthemes ‘Learning as a catalyst for change’ and ‘The need for institutional support’.

**CONCLUSIONS:**

The results reveal the need to integrate content about planned home births into midwifery curricula. High-fidelity simulation can help bring planned home birth to midwifery students and midwives with a dynamic, safe, and reflective approach, ensuring optimal care for women who choose a planned home birth. Additionally, the results identify resources that could promote the visibility of planned home birth attention within academic, clinical, and social environment.

## INTRODUCTION

In developed countries, approximately 11 million births were recorded in the past year^1^. Of these, 98% occurred in hospital^2^. In Spain, the number of births in 2024 was 322034^3^. Most births were assisted in delivery, with estimates exceeding 99%^4^. Recently, there has been an increase in the number of women seeking maternity care outside the hospital and choosing planned home birth^5^. Planned home birth refers to births assisted by midwives that take place at home in a planned setting, as chosen by the women and her partner^6^. In Spain, a midwife is a qualified professional who completes a midwifery education program that meets the standards of the International Confederation of Midwives, with a duration of two years after passing a competitive examination, and is registered in the country in order to develop competencies in midwifery practice focused on the care and support of women during pregnancy, labor, the puerperium, and the postnatal period, and their babies^7^. In Spain, midwives who attend planned home births work independently; home birth is not funded by the Public Health System (PHS)^8^. In Spain, the rate of planned home births is 0.4%, which is low compared to other high-income countries where planned home birth is funded by the PHS^9^. Among high-income countries, the Netherlands has the highest rate of planned home births, with approximately 16.3% of women choosing a planned home birth that is funded by the PHS^10^. The National Institute for Health and Care Excellence (NICE) recently published clinical practice guidelines to offer and support women to choose any community birth (delivery, birth center, or home) and stated that healthcare professionals involved in the process should support this decision^11^. Other international organizations support planned home birth as a safe option for low-risk women, such as American College of Obstetricians and Gynecologists^12^, and the International Confederation of Midwives^13^, which establishes clinical practice guidelines for the development of planned home births and highlights the importance of healthcare professionals providing information to women so that they can make a free and informed decision. The Federation of Midwives Associations of Spain (FAME) supports planned home birth as a safe option for low-risk women, upholding the right of women to make an informed decision about where to give birth, and promotes funding for this by the PHS, although no policy changes have been achieved to date^14^.

There is extensive evidence supporting the safety of planned home births for low-risk women^5,15^. Specifically, women who choose home birth are more likely to have a spontaneous birth compared to those who opt for a hospital setting^8^. Furthermore, women receive fewer iatrogenic interventions, such as the use of oxytocin, epidural anesthesia, or instrumental delivery^16^. Women experience fewer complications, such as infections, postpartum hemorrhage, or third- and fourth-degree perineal tears, and neonatal morbidity and mortality are comparable to hospital^15^. For these reasons, a small but relatively stable number of women choose to give birth at home^17^. However, opinions about planned home birth care among healthcare professionals remain polarized in many countries where this service is not publicly funded by PHS^8,18^. A study conducted in the United Kingdom reported that midwives were in favor of offering universal access to home birth care, while nurse practitioner and obstetricians expressed neutral views that could negatively influence women’s decisions to choose home birth^19^. Neonatologists have expressed negative opinions about home birth^6^. In the United States, a qualitative study reported that healthcare professionals did not recommend planned home birth, despite supporting women’s right to choose their place of birth^20^. Attitudes and levels of knowledge among different healthcare professionals regarding planned home births suggest that it remains a controversial topic and that further education is needed^21^.

Education and previous experiences of midwifery with regard to planned home births are decisive in shaping attitudes toward them^6^. Recent studies suggest that many healthcare professionals lack the knowledge and education required to adequately inform women about birth setting options^20,21^. Specifically, they are unfamiliar with what planned home birth care entails and the safety of this birth setting^6,21^. Midwifery students, as future midwives must acquire the necessary competencies to fulfill their responsibilities in providing care to women^18^. In this regard, community birth is included in the midwifery curriculum; however, planned home births are not addressed in most countries whose care is not funded by the PHS. The lack of funding from the PHS, the infrequency of this practice among women, and the influence of cultural factors further limit the knowledge of midwifery students about this birth setting^22^. Education of future midwives in home birth care is essential to promote visibility of this birth setting as a safe option^13^. Therefore, educating future midwife on this topic could help address awareness gaps and support the visibility of this birth environment. In this regard, high-fidelity simulation is an effective educational methodology for acquiring knowledge, developing clinical skills, and transferring them into obstetric clinical practice^23^. High-fidelity clinical simulation allows the integration of theoretical knowledge and critical thinking and promotes clinical decision-making, preparing students to attend to the needs of women^24^, implement the clinical practice guidelines established by various organizations for planned home birth care and increase their awareness of this issue^11-13^. However, previous research has not explicitly explored the experiences of midwifery students in planned home birth care to understand their perceptions and identify barriers to improving care for women in planned home births. Therefore, the aim of this study was to explore midwifery students’ experiences of clinical training in planned home birth and how to improve the quality of care based on identified barriers.

## METHODS

### Study design

This study adopted a descriptive qualitative research design^25^ based on semi-structured interviews on midwifery students’ experiences with a simulated planned home birth of a woman. Qualitative descriptive research is appropriate when seeking a comprehensive and direct representation of a phenomenon of interest, especially in situations that require naturalistic inquiry, such as the experiences of midwifery students who attended a planned home birth of a low-risk woman and their perspectives on planned home births^26^.

### Participants and setting

This study was conducted from December to April 2025. The study was carried out on midwifery students. In Spain, upon completion of their university nursing education, nurses may take part in a competitive entrance examination and if they pass, they gain access to education programs for midwifery. During this education program, midwifery students complete a two-year theoretical and practical education in the PHS. Upon completion, they must register as qualified midwives in order to work as midwives in both the public and private settings^7^. The inclusion criteria were: 1) be midwifery students in Spain, and 2) voluntarily agreeing to participate and be recorded. The exclusion criteria were: 1) being an exchange student without native-level Spanish language competence, and 2) having attended planned home births. The lead researcher contacted the participants via email, informing them of the opportunity to voluntarily participate in the study. A total of 13 midwifery students participated.

### Clinical simulation

**Figure 1:**
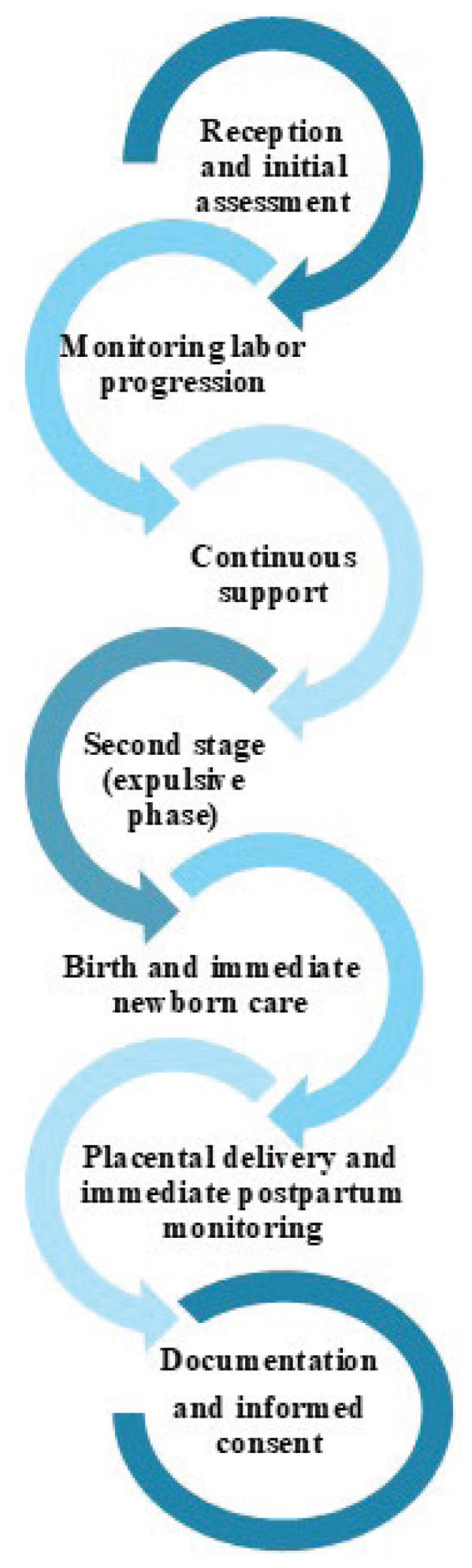
Protocol of care followed during the high-fidelity simulation of a planned home birth (sequence of actions expected from midwifery students during the scenario)

A high-fidelity clinical simulation was conducted with a standardized patient being a trained actress simulating a low-risk woman and a high-fidelity obstetric simulator (PROMPT Flex^®^, Laerdal Medical) used to recreate the scenario of a low-risk planned home birth, lasting approximately one hour and thirty minutes. The simulation, based on the care of a planned home birth, was set in a private home where a standardized patient simulating a low-risk woman had decided to give birth at home, accompanied by her husband. The home was located near the city’s referral hospital. The aim of this simulation was to introduce midwifery students to home births in lowrisk women as a safe clinical practice, to identify signs and symptoms of obstetric emergencies, and to work in coordination with other healthcare services. During planned home birth, students were required to follow international clinical practice guidelines for attending planned home births^11-13^. The simulation included a brief introduction for participants (approximately 10 minutes), in which they were informed about the scenario, setting, and learning objectives. This was followed by the simulation experience (approximately 30 to 40 minutes) and a final debriefing session (approximately 50 minutes) with the student who participated. In the simulation, midwifery students attended to a standardized patient simulating a low-risk woman who had decided to give birth at home, accompanied by her husband. The midwifery students had to attend to the low-risk woman in labor based on the Protocol of care of a planned home birth developed to follow international clinical practice guidelines for attending planned home births (Figure 1)^11-13^. The simulation was designed and conducted by registered midwives with more than five years of clinical experience, who support the implementation of planned home births as a birth setting.

### Data collection

The students were invited to participate voluntarily via email, which outlined the voluntary nature of the study, its objective, and the assurances of confidentiality and anonymous data processing. Students interested in participating contacted the principal investigator via email. Subsequently, students interested in participating were contacted via the same email address and invited to attend an in-person session. The session was held in a simulation seminar room of the Faculty of Health Sciences at University of Almeria. Data collection was carried out through 13 semi-structured interviews. Before starting data collection, an interview protocol was developed based on scientific literature, including the most relevant topics for the research (Supplementary file Table S1)^6,17,20^. After the simulation, interviews were conducted, each lasting between 40 to 60 minutes. The audio recordings were transcribed verbatim within 24 hours of each interview. All of the interviews were anonymized, using the letters ‘P’ (participant) followed by the participant number. Before data analysis, the transcripts were returned to the participants for verification to ensure their accuracy.

### Data analysis

**Figure 2:**
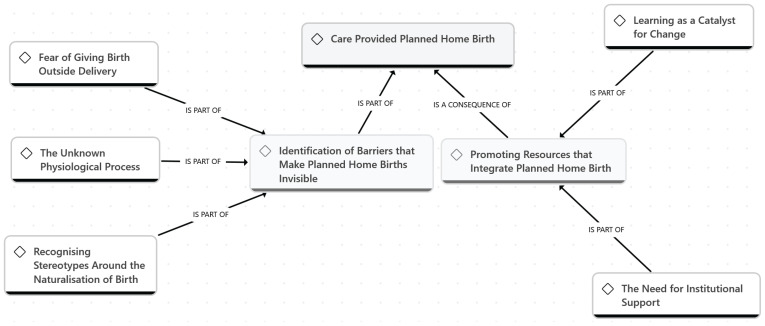
Conceptual map based on midwifery student’s experiences for planned home birth

The interviews recordings were transcribed verbatim. Subsequently, alphanumeric codes were assigned to each participant (P1, P2, etc.) to ensure confidentiality and anonymity. ATLAS.ti version 25.0.1 (ATLAS.ti Scientific Software Development) was used as a resource for data analysis. A reflexive thematic analysis was conducted following the phases described by Braun and Clarke^27^: 1) The data analysis and familiarization phase began during the transcription and initial reading of the texts, noting preliminary ideas; 2) Systematic data coding involved rereading the transcripts and generating initial codes across the dataset, which were then grouped by meaning and patterns; 3) Theme generation consisted of identifying preliminary themes and developing a thematic map to establish relationships among them; 4) Review and refinement of themes ensured consistency between codes and emerging themes; 5) Refinement of the analysis included finalizing theme names, definitions and the coding structure. The first and last authors preliminary themes were discussed; and 6) The writing of the report included the analysis of previously selected extracts and the link of the analysis to the research question for the final interpretation. A conceptual map was developed to illustrate the main themes reflecting the experiences and perceptions of midwifery students about planned home births (Figure 2).

### Reflexivity and trustworthiness

The validity, reliability, confirmability, and transferability of the study were ensured by applying various techniques. Specifically, the data were translated by one bilingual researcher to English. Then, another bilingual researcher back-translated them to Spanish and compared with the original transcripts to ensure reliability^25^. Two researchers independently analyzed the interviews following the guidelines established by Braun and Clarke^27^ to ensure the validity and accuracy of the data. Discrepancies related to study design, data analysis, and conclusions were discussed among the members of the research team until consensus was reached^28^. The researchers provided participants with transcripts that included the codes assigned in the analysis to allow verification, thus ensuring confirmability^25^. Participants confirmed the accuracy of the transcripts, and no changes were made after verification. Regarding transferability, a detailed description of the setting, participants, context, and method was provided to serve as a reference for future studies^26^.

### Ethical considerations

The study received low risk approval from the Ethics Committee and Research of Nursing, Physiotherapy and Medicine of the University of Almeria (EFM 354.24, Date of approval: 28/10/2024). This study adhered to the principles outlined in the Declaration of Helsinki. The standardized patient was a trained actress simulating a low-risk pregnant woman who signed an informed consent form after being informed of the purpose of the study and its voluntary nature, also specified that no procedures or situations that could pose any risk to her health would be performed. The study was considered low risk for participants as the high-fidelity simulation was conducted in a laboratory at the Faculty of Health Sciences at the University of Almeria and participants only had to undergo interviews about their experience. Before beginning the high-fidelity simulation, participants were verbally informed of the voluntary nature of their participation, the objective of the study, the anonymous and confidential treatment of data, and the possibility of withdrawing from the study at any time. Participants understood that participating in the study would not affect their academic grades. There was no academic relationship between the researchers and the participants that could have influenced their decision to participate. To mitigate interviewer bias, the interviewers contacted by the principal investigator did not work as preceptors of midwifery students. Before their participation, all students provided informed consent by signing a form indicating their voluntary participation in the study. The audio recording and transcription were stored to Google Drive, ensuring access to only to the researchers who analyzed the data.

## RESULTS

A total of 13 interviews were conducted. The average age of midwifery students was 27.84 years (SD = 3.46) ranging from 23 to 35 years. Overall, 76.9% of the participants (n=10) were women and 23.1% (n=3) were men. Among midwifery students, 61.5% (n=8) were in the first year while 38.5% (n=5) were in the second year of education for midwifery. The analysis identified two main themes: 1) ‘Attend a planned home birth’ and 2) ‘Promoting resources that integrate planned home birth’ (Figure 2).

### Identification of barriers that make planned home births invisible

This theme includes three sub-themes that reflect participants’ perceptions during their first experience attending a planned home birth through clinical simulation. This experience identified emotional, academic, institutional, and cultural difficulties that affect safe care in planned home births. Moreover, the simulation helped them understand how to offer quality care and provide women with adequate information, enabling them to make informed decisions about their birthing preferences.

### Fear of giving birth outside delivery

Attending a birth in an out-of-hospital setting, without prior experience and without the support of hospital resources, generated fear, insecurity, and stress due to the perception of the setting as unsafe and the possibility of complications arising. Furthermore, the participants experienced difficulties in decision-making, acknowledging that these personal and emotional barriers interfered with their performance in providing safe care during the planned home birth attended:

*‘It is very stressful to think that, at any moment, something could go wrong. Just imagine! You are outside the hospital, alone, without resources ... that’s why I think the hospital is safer.’* (P3)

*‘The problem was that, with the nerves and my lack of knowledge, I froze and found it hard to make decisions ... the situation was overwhelming for me.’* (P2)

### The unknown physiological process

In addition, a large number of participants mentioned the limited specific education they had received about home birth, highlighting the importance of understanding key aspects to identify, assess, and manage potential complications that can arise during birth in this context. The simulation helped them gain a better understanding in order to provide quality care and appropriate information to woman, enabling them to make informed decisions about their preferred community birth:

*‘We have worked on delivery, but nothing at all about planned home births. We need more information and education that helps us know how to respond, especially if the situation becomes complicated ... I have only seen what we did in this simulation, which was great. Now, I see myself responding differently if the same situation happens again.’* (P8)

### Recognizing stereotypes around the naturalization of birth

Most of the participants believed that only a delivery provided the appropriate conditions to attend a birth. This belief, coupled with limited institutional support, the lack of specific protocols and scarce logistic resources, generated a sense of vulnerability among participants, reinforced by their perception of their practice as marginal and risky, distancing childbirth from the physiological process:

*‘Giving birth at home can be risky for both mother and infant. If any problems arise, we would have to take her to the hospital. Who would take us there? Who would wait for us? My goodness, without protocols or support, the time it takes could be dangerous and mortal.’* (P11)

The participants confessed that their perspective on planned home birth was influenced by negative anecdotes, unconfirmed information, and a reluctance to stray from the dominant biomedical model in the current PHS. Likewise, some participants stressed the emphasized lack of culture surrounding this practice in both clinical and social environment, fostering erroneous preconceptions that stigmatized this place as unsafe, negatively affecting its inclusion in PHS:

*‘My opinion is that it is a risky situation, and I think this mainly because of what my colleagues say: that if the pain becomes unbearable, that dilation stops, that at any moment things can go wrong, a cord prolapses, a hemorrhage. Let’s face it, childbirth is not always so physiological!’* (P 7)

*‘I think professionals and people think it’s crazy, hugely irresponsible, so no one dares to do it.’* (P 8)

### Promoting resources that integrate planned home birth

This theme highlights the need to incorporate strategies that make planned home birth visible and integrated within academic, clinical, and social environments as a safe practice. This includes inclusion in the PHS, in the content of midwifery curricula, and the development and implementation of interventions in academic, clinical and social environments that enable the education programs for midwifery students and midwives, guaranteeing quality care and the right of women to freely choose where to give birth, as well as social acceptance.

### Learning as a catalyst for change

The analysis of the interviews revealed a lack of knowledge about planned home birth as one of the main factors increasing resistance to its implementation in clinical settings and its integration into the PHS. For the majority of participants, the need to include theoretical and practical content in midwifery curricula that guarantee accurate information to reinforce women’s autonomy in decisionmaking and quality care in planned home births was highlighted. Additionally, they stated that the simulation changed their preconceived ideas and that they perceived the autonomy and competencies of midwives:

*‘In my opinion, home birth is a reality and if a woman wishes to give birth at home, it is legitimate and we must respect her decision and inform her, since that’s what we are here for. However, we must first educate ourselves, since, at present, our knowledge is non-existent.’* (P12)

*‘This experience has really surprised me. I have realized that planned home births highlight our autonomy as midwives.’* (P10)

### The need for institutional support

The support of government and healthcare institutions is essential to promote the visibility of planned home birth as a safe option, to ensure that women can freely decide and to bring about cultural change. Some participants emphasized that the development and implementation of strategies promoted by healthcare managers, such as the implementation of clinical practice guidelines, the provision of resources, the continuous education of midwifery, and the inclusion on education program of midwifery students, would contribute to professional recognition and reduce variability in this practice, thereby supporting its legitimization and normalization within PHS. Additionally, the participants highlighted that dissemination strategies through various media platforms would help increase public awareness and bring this birth setting closer to society:

*‘I think managers and hospital staff should guarantee the necessary resources to assist her when she wants it ... It is essential to have very clear protocols so that all professionals know what to do at all times and that the woman feels safe.’* (P5)

*‘For me, for example, it would be essential to have an ambulance very close to home in case of any complications and a hospital team on standby and ready to receive you in case of urgent transfer.’* (P4)

*‘It is necessary for all health care professionals and society in general to be aware of this, to see it as something normal. Campaigns should be carried out, using social media, the media, health centers, schools, etc.’* (P13)

## DISCUSSION

The aim of this study was to explore midwifery students’ experiences of clinical training in planned home birth and how to improve the quality of care based on identified barriers. According to the research findings, the first experience with planned home birth of midwifery students provided a comprehensive and practical perspective on the topic, identifying personal, academic, institutional, and cultural factors that influence midwifery students’ attitudes towards planned home births. Furthermore, it highlights the need to promote the development of strategies from institutions to integrate planned home births and make them more visible, so that women can freely choose the place in which they wish to give birth. The findings of this study reflect that the lack of specific training in planned home births negatively affects midwifery students’ perceived safety and self-confidence in providing safe, quality care to women who choose this option. This educational gap, associated with the medicalization of childbirth in countries such as Spain, has contributed to shifting the physiological and natural view of birth towards a more interventionist perspective, generating insecurity and feelings of fear when students work out of hospital settings. Consistent with data obtained in previous studies, the lack of clinical experience in planned home births generates uncertainty and fear in students, decreasing their self-confidence and increasing their perception of risk when caring for women^6,28^. Previous research highlights that prior experience and specific training in community births for midwifery students boosts confidence, reduces stress and increases their ability to manage potential emergencies, thus establishing a safe model of birth care^29,30^. In this regard, exposing midwifery students to rare situations through clinical simulations allows them to develop critical thinking and overcome fears about out-of-hospital births^13^. High-fidelity simulation, such as that employed in this study, enables midwifery students to experience unusual clinical settings and enhances their confidence and ability to respond to complications^6,23^. In the Spanish context, where home births are neither funded nor common, these educational strategies may be crucial in enabling future midwives to acquire knowledge and confidence in caring for low-risk women who choose this setting.

The results of this study show that cultural and professional prejudices towards planned home births influence midwifery students’ perceptions, generating insecurity and opposition to the development of this practice, coinciding with data reported in previous studies^18,22^. This perception of planned home births among midwifery students was triggered by low social visibility, the training received, and the predominant medicalized culture of childbirth they experience during their clinical practice, reiterating that the hospital is the only safe environment for women and their babies. In this regard, a recent study shows that a lack of knowledge about the safety and scientific evidence of planned home births encourages the development of negative perceptions among midwifery students and midwives^23,24^. Furthermore, the medicalized culture of childbirth present in clinical training in countries where planned home births are not funded by the PHS, conditions the attitudes of students, promoting an interventionist and hospital-dependent view^31,32^. Training future midwives for planned home births is essential to ensure quality care and reduce complications for both women and babies^28,29^. In this regard, high-fidelity clinical simulation has been shown to be an effective learning methodology for midwifery students to acquire clinical skills and a humanized, physiological and safe understanding of planned home births^29,33^. Therefore, incorporating evidence-based content and simulation experiences into midwifery programs can help transform these perceptions and promote understanding of planned home births.

The data from this study revealed the need to integrate resources and strategies in both academic and clinical settings to ensure adequate training for midwifery students in planned home birth care. High-fidelity clinical simulation allowed students to learn about home birth as a safe option and identify strategies such as the inclusion of specific content in training programs, the use of clinical simulation, and continuing education for midwives to ensure the acquisition of skills, increased self-confidence, and professional autonomy. Along the same lines, several studies highlight the need to include specific training in obstetrics programs to overcome perceptual barriers related to home birth and acquire knowledge and skills related to physiological birth^18,24^. Personal and academic experiences shape attitudes towards planned home birth, and exposure to planned home birth during education for midwifery are key to fostering positive attitudes toward this practice^6^. In this regard, the simulation allowed students to approach birth attendance as a safe practice, identify the needs of women during labor, and recognize the essential elements required to provide high-quality care. High-fidelity simulation has proven to be an effective methodology to acquire new skills and knowledge in obstetrics, management obstetric emergencies, and transferring learning to clinical practice^23,24^. This methodology may have a crucial role in helping both midwifery students and midwives become familiar with other community birth as a planned home birth, promoting childbirth as a physiological process, and acquiring the necessary skills to provide information and support for planned home births efficiently, as well as to increase the visibility of this practice.

This study emphasizes that the availability of resources is a determining factor for the integration of planned home births into the PHS. The absence of standardized protocols regulating planned home birth care and adequate equipment limits safety and continuity of care in Spain. Institutional support is essential to ensure the visibility and integration of planned home births in both clinical and academic settings, promoting the professional autonomy of midwives. In this sense, the integration of planned home births into PHS helps reduce inequalities, normalize practice, and eliminate barriers such as social and professional stigma. Sanchez-Redondo et al.^34^ affirm that home birth can offer benefits in well-structured contexts, but Spain does not provide the material resources or a PHS prepared to guarantee maternal and infant safety in this setting of birth. However, in countries where planned home births are funded by the PHS, effective coordination between different levels of care is guaranteed in the event of transfer during labor, as is ongoing education for midwives^8,31^ and equitable access to the home birth^16^ respecting women’s right to decide where they want to give birth. Several studies highlight that the availability of adequate resources such as updated protocols, inclusion of content in the curriculum, simulation rooms, and institutional support would enable midwifery students to be prepared for safe care during planned home births, promoting a physiological approach to childbirth^35,36^. Nurse managers and healthcare administrators were identified as key agents of change for the professional development of midwives and other healthcare professionals involved in childbirth, ensuring adequate training for planned home births. Education programs for midwives and other healthcare professionals involved in childbirth about planned home births would support midwives’ professional practice in home births^18,33^. They highlighted that including planned home births within the PHS would allow this practice to be carried out safely and enhance its visibility.

### Limitations

This qualitative descriptive study provides valuable information and perspectives from midwifery students on planned home births. However, several limitations should be considered. First, to our knowledge, no study addresses the use of high-fidelity clinical simulation for planned home birth education midwifery students, which limits the depth of discussion of the results. On the other hand, the participants came from one country, which means that students from other countries may have different experiences and opinions about planned home births, potentially limiting the transferability of the findings. Diversifying study settings could improve the comprehensiveness and applicability of research results in different contexts. Future research should further explore the experiences and perceptions of midwives, both with and without prior experience in attending planned home births, in order to better understand the home birth process and promote its acceptance among midwives and other healthcare professionals.

## CONCLUSIONS

This study highlights the personal, emotional, educational, and cultural factors that hinder quality of care at planned home births. The findings reveal the need to integrate content related to planned home births into midwifery curricula. High-fidelity simulation can help bring planned home birth closer to midwifery students and midwives through a dynamic, safe, and reflective approach, ensuring optimal care for women who choose this environment of birth. The data suggest that midwifery educators and registered midwives, as well as government and healthcare managers, should develop strategies to raise awareness of this model of care in academic, clinical and social settings, to promote midwife autonomy, regulation of this practice, women’s freedom to choose the place of birth they want, and demystify social stereotypes.

## Supplementary Material



## Data Availability

The data supporting this research are available from the corresponding author on reasonable request.
